# Excluding Digenic Inheritance of *PGAP2* and *PGAP3* Variants in Mabry Syndrome (OMIM 239300) Patient: Phenotypic Spectrum Associated with *PGAP2* Gene Variants in Hyperphosphatasia with Mental Retardation Syndrome-3 (HPMRS3)

**DOI:** 10.3390/genes14020359

**Published:** 2023-01-30

**Authors:** Miles D. Thompson, Xueying Li, Michele Spencer-Manzon, Danielle M. Andrade, Yoshiko Murakami, Taroh Kinoshita, Thomas O. Carpenter

**Affiliations:** 1 Adult Genetic Epilepsy (AGE) Program, Toronto Western Hospital, Krembil Brain Institute, Toronto, ON M5T, Canada; 2 Osaka University, 3-1 Yamada-Oka, Osaka 565-0871, Japan; 3 Departments of Genetics and Pediatrics, Yale University, New Haven, CT 06520, USA; 4 Division of Neurology, Department of Medicine, University of Toronto, Toronto, ON M5S, Canada; 5 Yale Pediatrics (Endocrinology), Yale University School of Medicine, New Haven, CT 06521, USA

**Keywords:** developmental disability, glycosylphosphatidylinositol (GPI) disorder, Mabry syndrome, hyperphosphatasia with mental retardation (HPMRS), exome panel sequencing, post attachment to proteins, type 2 (PGAP2), rescue assay, flow cytometry, CD55, CD59

## Abstract

We present a case report of a child with features of hyperphosphatasia with neurologic deficit (HPMRS) or Mabry syndrome (MIM 239300) with variants of unknown significance in two post-GPI attachments to proteins genes, *PGAP2* and *PGAP3*, that underlie HPMRS 3 and 4. Background: In addition to HPMRS 3 and 4, disruption of four phosphatidylinositol glycan (PIG) biosynthesis genes, *PIGV*, *PIGO*, *PIGW* and *PIGY*, result in HPMRS 1, 2, 5 and 6, respectively. Methods: Targeted exome panel sequencing identified homozygous variants of unknown significance (VUS) in *PGAP2* c:284A>G and *PGAP3* c:259G>A. To assay the pathogenicity of these variants, we conducted a rescue assay in *PGAP2* and *PGAP3* deficient CHO cell lines. Results: Using a strong (pME) promoter, the *PGAP2* variant did not rescue activity in CHO cells and the protein was not detected. Flow cytometric analysis showed that CD59 and CD55 expression on the PGAP2 deficient cell line was not restored by variant *PGAP2*. By contrast, activity of the *PGAP3* variant was similar to wild-type. Conclusions: For this patient with Mabry syndrome, the phenotype is likely to be predominantly HPMRS3: resulting from autosomal recessive inheritance of NM_001256240.2 *PGAP2* c:284A>G, p.Tyr95Cys. We discuss strategies for establishing evidence for putative digenic inheritance in GPI deficiency disorders.

## 1. Introduction

Hyperphosphatasia with neurologic deficit (MIM 239300), Mabry syndrome (HPMRS), manifests in the first year of life. The phenotype includes three cardinal features: developmental disability, seizures, hyperphosphatasia [1] with or without brachytelephalangy. Traditionally, elevation of tissue non-specific alkaline phosphatase served to distinguish HPMRS from other intellectual disabilities, especially those with similar facial anomalies: wide set eyes, up-slanting palpebral fissures, short nose with broad nasal bridge and tip and tented upper lip [2,3]. While analysis with human phenotype ontology (HPO) has shown that hyperphosphatasia itself is not indicative of severity [4], it is still necessary for a clinical HPMRS diagnosis [2].

Since the identification of biallelic variants of the phosphatidylinositol glycan (PIG), type V (*PIGV*) gene in HPMRS [5], the disorder has been known to be one of at least 21 inherited glycosylphosphatidylinositol (GPI) deficiencies (IGD). These congenital disorders of glycosylation result from disruption to at least 16 of the 27 genes encoding GPI biosynthesis, bringing about GPI biosynthesis deficiency (GPIBD) [6,7,8]. The GPIBDs result from biallelic inheritance of variants in genes encoding proteins participating in the GPI) biosynthesis and remodeling pathway [2,6,9], comprising the PIG biosynthesis pathway and the post-GPI-attachment-to-proteins (PGAP) remodeling process [10].

Since GPI-anchored proteins (GPI-APs) include hydrolytic enzymes, adhesion molecules, receptors, protease inhibitors and regulatory proteins in the complement pathway, the phenotypes resulting from their disruption are quite broad [11]. The GPIBDs share many features [12] such as global developmental delay, intellectual disability, hypotonia, seizures, and facial dysmorphisms. In addition to the hyperphosphatasia characteristic of HPMRS, the syndrome can feature hypoplastic nails, and brachytelephalangy [3,11]. Microcephaly, hearing impairment, joint contractures, skin anomalies, congenital heart defects, urinary tract defects, and skeletal anomalies may be present in some patients [3].

Building on the original syndromology of Mabry et al. (1970) [1], subsequently corroborated by others [2,13,14,15], whole exome and genome sequencing has identified recessive mutations in at least six genes that can independently result in six HPMRS phenotypes, HPMRS1–6 [3,6,9]. Bi-allelic inheritance of four genes expressed in the endoplasmic reticulum (ER) have been reported to result in the HPMRS phenotype [3,6,9]. Numbered in order of discovery, these include *PIGV* variants resulting in HPMRS1, or GPIBD2 (MIM: 239300) [5], HPMRS2 (MIM: 614749), or GPIBD6, resulting from *PIGO* variants [16,17,18]; HPMRS5 (MIM: 616025), or GPIBD11, resulting from *PIGW* variants [19] and HPMRS6 (MIM: 616809), or GPIBD12, resulting from *PIGY* variants [20].

HPMRS phenotypes can also result from bi-allelic inheritance of variants in two genes encoding post-GPI attachment to proteins (PGAP) that, expressed in the Golgi, help, to stabilize membrane attachment of GPI-anchored proteins (GPI-AP): HPMRS3 (MIM: 614207), or GPIBD8, resulting from *PGAP2* variants [21,22,23,24] and HPMRS4 (MIM: 615716), or GPIBD10, resulting from *PGAP3* variants [25,26] (https://ncbi.nlm.nih.gov/gtr/conditions/C1853205/) (accessed on 1 December 2022).

Since our recent 50-year follow-up [27] of Dr. Mabry’s original patients [1], which identified novel bi-allelic variants of *PGAP2* associated with the original HPMRS phenotype [28], we have continued to work with individuals whose HPMRS phenotype cannot be explained by known gene variants. In the present case report, we describe the phenotype of a severely affected child with Mabry syndrome found to have inherited biallelic variants of unknown significance (VUS) in both *PGAP2* and *PGAP3*. Since SIFT and PolyPhen modelling was inconclusive with respect to identifying the most likely pathogenic variant, we could not exclude digenic inheritance until the in vitro work presented here was performed.

## 2. Case Report

A male child of 2 years of age was referred to the Yale Pediatric Specialty Center with severe developmental delay and autistic features (summarized in Table 1).

While he had subtle features of Mabry syndrome, predominantly the short nose with broad tip, there were few other signs but morphologically. The alkaline phosphatase levels were elevated at 3–4 times the upper limit of normal. There was a history of distant consanguinity.

## 3. Materials and Methods

### 3.1. Molecular Genetics

Chromosome microarray (Yale) analysis was conducted to identify larger chromosomal rearrangements such as deletions, insertions, inversions and other copy number variations (CNVs).

### 3.2. Targeted Exome Panel Sequencing

The Prevention Genetics autism spectrum disorder (ASD) panel was run at Prevention Genetics, 3800 South Business Park Ave. Marshfield, WI, 54449, USA, on 31 October 2017. This panel typically provides 99.1% coverage of all coding exons of 170 genes plus 10 bases of flanking noncoding DNA in all available transcripts along with other non-coding regions in which pathogenic variants have been identified at Prevention Genetics or reported elsewhere.

https://www.preventiongenetics.com/testInfo?val=Autism-Spectrum-Disorders-%28ASD%29-Panel (accessed on 1 December 2022).

### 3.3. In Vitro Rescue Assays

A rescue assay, involving the expression of the *PGAP2* c:284A>G variants in *PGAP2* [29] deficient CHO cells was conducted to determine the potential of the variant for pathogenicity. The variant was expressed in the context of PGAP2 transcript 12, NM_001256240.2 *PGAP2* c:284A>G, p.Tyr95Cys, encoding isoform 8, since isoform 1 is inactive [28].

**Figure 1:**
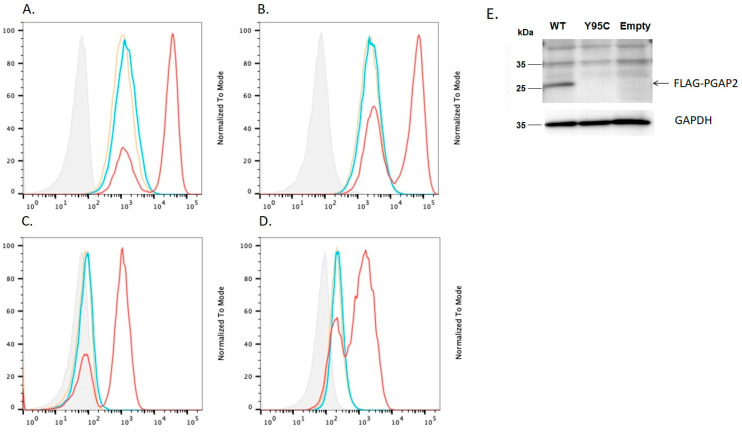
The effect of c.284A>G mutant *hPGAP2* on the expression of GPI-anchored proteins (GPI-APs) in *PGAP2* deficient CHO cells. Isotype (grey); PME WT FLAG-*hPGAP2* (red); PME c.284A>G mutant FLAG-*hPGAP2* (blue); PME empty (yellow). Compared with wide type *hPGAP2*, *hPGAP2* c.284A>G could not rescue expression of GPI-APs, (**A**). CD59; (**B**). CD55; (**C**). uPAR; (**D**). FLAER in *PGAP2* deficient CHO cells. (**E**). Western blot analysis of the FLAG *hPGAP2* proteins in CHO cells. The result showed that hPGAP2 mutant protein (p.Try95Cys) was not detected, while the wild-type hPGAP2 protein was detected at 26 kDa by the anti-FLAG antibody. The mutation destabilized the PGAP2 protein expression.

FLAG-tag was attached to the amino-terminus of PGAP2, as described previously [28]. Two days after having transfected wild type or mutant PGAP2 cDNA, driven by strong pME promoters into the PGAP2 deficient CHO cells, fluorescence-activated single cell sorting (FACS) analysis (Figure 1A–D) and western blot analysis (Figure 1E) with anti-FLAG antibody were performed. 

A rescue assay, involving the expression of the C-terminal HA-tagged *PGAP3* c:259G>A driven by the pME promoter in *PGAP2* and *PGAP3* [30] double deficient CHO cells, was conducted to determine the potential of the variant for pathogenicity. Thus, the proxy of the phenotype was taken to be the relative inability of a variant construct to rescue complement decay-accelerating factor (CD55), CD59 glycoprotein (CD59), Urokinase Plasminogen Activator Receptor (uPAR) and Fluorescein-labeled proaerolysin (FLAER) compared with wild-type, as measured by flow cytometry. Subsequently, the pME prompter was replaced with the weaker pCMV, in order to see the difference in the degree of GPI-APs expression more clearly.

## 4. Results

### 4.1. Molecular Genetics

Chromosome microarray (Yale) analysis revealed an XY male with a 58 Kb deletion at 2p13.1 (chr2:73,853,219-73,911,035, including the *NAT8* (N-Acetyltransferase 8) and *ALMS1P* (ALMS1 Centrosome And Basal Body Associated Protein) genes) and a 109 Kb duplication at 11q13.5 (chr11:75,237,557-75,347,017, including the *SERPINH1* (Serpin Family H Member 1) and *MAP6* (Microtubule Associated Protein 6) genes).

Deletions and duplications at 2p13.1 have been documented in the Database of Genomic Variants (DGV, http://projects.tcga.ca/variation/) (accessed on 1 December 2022). The 58 Kb deletion at 2p13.1 observed in this patient is a likely benign copy number variant (CNV).

The duplication at 11q13.5 is not seen in the DGV. Mutations in the *SERPINH1* gene cause autosomal recessive osteogenesis imperfecta type X (OMIM#613848) (accessed on 1 December 2022). The duplication at 11q13.5 observed in this patient is considered to be a variant of uncertain significance (VUS).

### 4.2. Sequencing

Results of molecular investigation conducted on the Prevention Genetics ASD-ID panel are as follows:

Coding variants were identified in: AT-hook DNA binding motif containing 1 (*AHDC1*) c.4178 G>A; VUS in Xia–Gibbs Syndrome; Dihydropyrimidine (DPYD) c.257C>Y; Likely pathogenic in dehydrogenase deficiency/5-FU toxicity); Pyruvate Dehydrogenase (*DLAT*) c.32A>C. VUS in Pyruvate Dehydrogenase E2 deficiency. Most of the heterozygote variants appear to be less meaningful.

While a VUS was identified in Zinc Finger MYND-Type Containing 11 (*ZMYND11*) c.1378 G>A, the variant was not considered likely to be pathogenic for the associated autosomal dominant mental retardation. 

Bi-allelic VUS in *PGAP2* (NM_001256240.2 c.284A>G, p.Tyr95Cys) and *PGAP3* (NM_033419.3 c.259G>A, p.Val87Met) were identified, however, that suggested a possible molecular diagnosis of hyperphosphatasia with MR syndrome type 3, HPMR3 and/or hyperphosphatasia with MR syndrome type 4, HPMRS4, respectively. 

Further investigations included parental testing. The panel was used to identify the following gene variants in the parents. The mother is heterozygous for the *AHDC1* variant, the *PGAP2* variant and the *PGAP3* variant. The father is heterozygous for the *DLAT* variant, the *ZMYND11* variant and both the *PGAP2* variant and *PGAP3* variant.

### 4.3. In Vitro Rescue Assays

Mutant *PGAP2* cDNAs, driven by pME promoter, could not rescue the expression of CD59, CD55, uPAR and FLAER at all even with the strong promoter (pME)-driven construct (Figure 1A–D). Western blots showed mutant PGAP2 protein could not be detected, while the wild-type hPGAP2 protein was detected at 26 kDa by the anti-FLAG antibody (Figure 1E). 

To determine the functional consequences of c.259G>A *PGAP3* variant, we used mutant CHO cells defective in both PGAP3 and PGAP2. This is done because we cannot distinguish wild-type CHO cells and PGAP3-deficient CHO cells by flow cytometry analysis [31].

**Figure 2:**
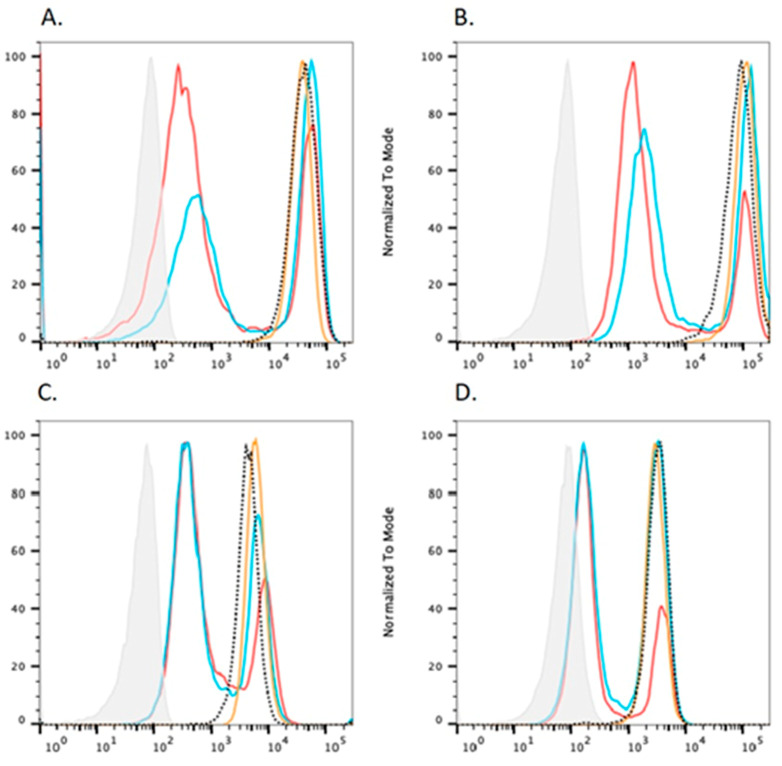
The effect of c.259G>A mutant *hPGAP3*, driven by the strong pME promoter, on the expression of GPI-APs in *PGAP2*- and *PGAP3*-deficient Chinese hamster ovary (CHO) cells. Isotype (grey); PME WT *hPGAP3*-HA (red); PME c.284A>G mutant *hPGAP3*-HA (blue); PME empty (yellow). The wide type *hPGAP3* and *hPGAP3* c.259G>A reduced the expression of GPI-APs to similar extents (**A**). CD59; (**B**). CD55; (**C**). uPAR; (**D**). FLAER in *PGAP2-* and *PGAP3-* double deficient CHO cells.

When wild-type *hPGAP3* cDNA was transfected into PGAP2/PGAP3-double-deficient CHO cells, the first step in the fatty acid remodeling was restored, whereas the second step remained defective, leading to the release of GPI-APs with only one fatty acid chain (i.e., becoming PGAP2-deficient cells). As shown in Figure 2, the activity of c.259G>A *hPGAP3* was similar to the wild-type *hPGAP3* when driven by a strong promoter, pME.

**Figure 3:**
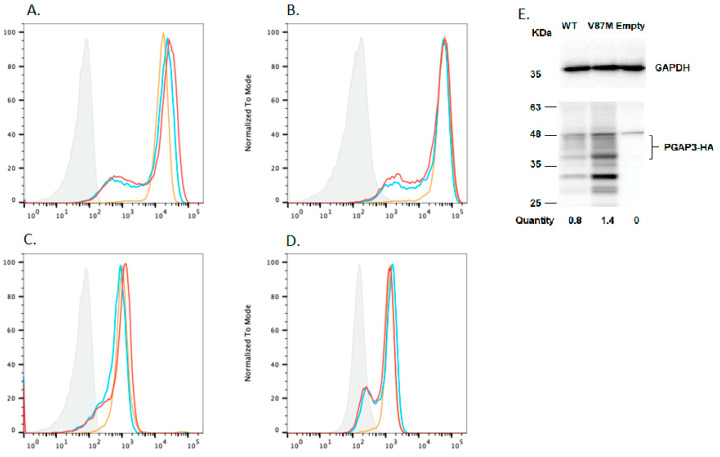
The effect of c.259G>A mutant *hPGAP3*, driven by the pCMV promoter, on the expression of GPI-APs in *PGAP2*- and *PGAP3*-deficient Chinese hamster ovary (CHO) cells. Isotype (grey); pCMV WT *hPGAP3*-HA (red); pCMV c.284A>G (p. Val87Met) mutant *hPGAP3*-HA (blue); pCMV empty (yellow). The wide type *hPGAP3* and *hPGAP3* c.259G>A reduced the expression of GPI-APs to similar extents (**A**). CD59; (**B**). CD55; (**C**). uPAR; (**D**). FLAER in *PGAP2-* and *PGAP3-* double deficient CHO cells. (**E**). Western blot analysis of the hPGAP3-HA proteins expressed in CHO cells by the pCMV promoter. The glycosylated PGAP3 proteins were detected by the anti-HA antibody. As for the quantity, each band intensity was normalized by that of GAPDH as a loading control and by luciferase activities as a transfection efficiency.

In order to determine the difference in the degree of GPI-AP expression more clearly, we repeated the FACS analysis (Figure 3A–D) having replaced the strong promoter pME with pCMV, which is weaker than the pME promoter. Having normalized variant and wild-type expression, we did not find evidence that this *hPGAP3* mutation affected its activity. 

## 5. Discussion

We report on the molecular diagnosis of a ten year-old boy with features of HPMRS who, in the course of gene panel sequencing, was found to have coding VUS in *PGAP2* and *PGAP3* genes. As a result, after coding variants in other genes were excluded from likely involvement in the HPMRS phenotype, we set out to classify the child as suffering HPMRS3, HPMRS4 or a novel digenic form of HPMRS.

In addition to the *PGAP2* and *PGAP3* variants, sequencing identified variants in five other genes. *AHDC1* c.4178 G>A, a VUS in autosomal recessive Xia-Gibbs Syndrome [31]; *ZMYND11* c.1378 G>A variant implicated in autosomal dominant mental retardation [32]; Dihydropyrimidine (*DPYD*) c.257C>Y, which is likely pathogenic in dehydrogenase deficiency/5-FU toxicity [33]; Pyruvate Dehydrogenase (*DLAT*) c.32A>C, a VUS in Pyruvate Dehydrogenase E2 deficiency [34]. 

These variants were excluded for the following reasons. The *DPYD* and *DLAT* variants were not considered to be relevant to HPMRS on the basis that, respectively, the phenotype of dehydrogenase deficiency/5-FU toxicity had no bearing on HPMRS and the Pyruvate Dehydrogenase E2 deficiency VUS was not only of doubtful significance and likely irrelevant to HPMRS. While *AHDC1* c.4178 G>A, a VUS in Xia-Gibbs Syndrome [31], a syndrome sharing some features with HPMRS, such as intellectual disability and hypotonia, it does not include the hyperphosphatasia characteristic of HPMRS noted in this patient. Although implicated in autosomal dominant mental retardation [34], the *ZMYND11* c.1378 G>A variant was not considered likely to be pathogenic. In addition, the 58 Kb deletion at 2p13.1 (chr2:73,853,219-73,911,035, including the *NAT8* and *ALMS1P* genes) and a 109 Kb duplication at 11q13.5 (chr11:75,237,557-75,347,017, including the *SERPINH1* and *MAP6* genes), were not considered to be relevant to the phenotype.

**Figure 4:**
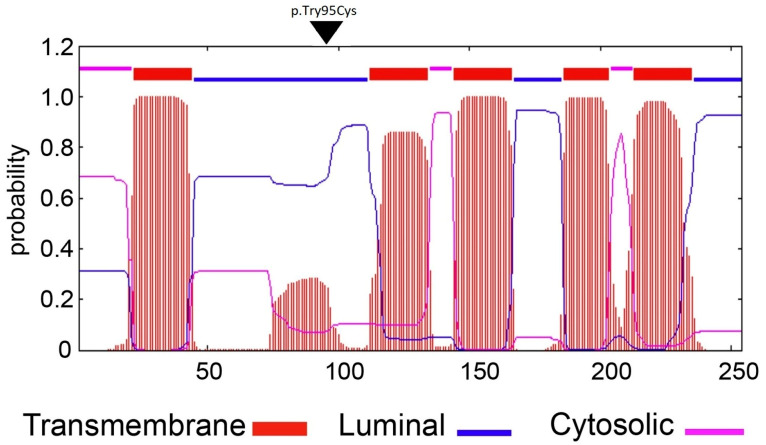
The likely pathogenic *PGAP2* variant is shown in context of the isoform 8 (arrow). *PGAP2* c.284A>G in the transcript 12, isoform 8 (NM_001256240) encoding p.Try95Cys was tested in the rescue assay. Transcript 1 encodes the less active isoform 1 whereas transcript 12, lacking 61 amino acids translated from exon 3, encodes the more active form as previously described [28]. The predicted topology of the PGAP2 protein was modeled using TMHMM (http://cbs.dtu.dk/services/TMHMM/) (accessed on 1 December 2022).

In order to determine whether the *PGAP2* or *PGAP3* variants are likely to be pathogenic, we assayed the extent to which both variants were able to rescue, respectively, the phenotype of PGAP2 deficient cells [29] and PGAP2/PGAP3 double deficient cells [30], as reported previously [28]. We used PGAP2 deficient CHO cells in order to assess the difference in PGAP2 function rescued by constructs encoding wild type versus mutant isoform 8. Compared to wild type, the *PGAP2* variant cDNA, driven by the strong pME promoter, could not rescue PGAP2 activity at all: as measured by FACS analysis of CD59 and CD55 (Figure 1). This suggests that the PGAP2 variant expressed in isoform 8 (Figure 4) is likely to be a non-functional variant.

By contrast, we found that compared to wild-type, the *PGAP3* c.259G>A construct, driven by the pME promoter, expressed in PGAP2/PGAP3 deficient CHO cells was able to rescue PGAP3 activity: as measured by FACS analysis of CD59 and CD55 (Figure 2). In order to control for the possibility that our results were specific to the strong pME promoter, we then used the weaker pCMV promoter to express the *PGAP3* c.259G>A variant construct. Compared to wild type, the *PGAP3* c.259G>A variant cDNA, driven by the weak pCMV promoter, was able to rescue PGAP3 activity at the similar level as wild-type: as measured by FACS analysis of CD59 and CD55 (Figure 3).

From these experiments, we conclude that NM_001256240.2:*PGAP2* c:284A>G, p.Tyr95Cys is more likely to be pathogenic, when inherited in the biallelic form, than the *PGAP3* c.259G>A variant. This interpretation is based on the results of our rescue assay, in which the expression of PGAP2 was markedly reduced compared with wild-type: a reduction which may impair GPI-anchor remodeling necessary for stable anchoring of GPI-APs, such as CD55 and CD59. We speculate that the missense mutation may result in an unstable PGAP2 protein, leading to decreased expression.

These data support the molecular diagnosis of the individual presented in this case study with HPMRS3: consistent with biallelic inheritance of a likely pathogenic *PGAP2* variant. Our work effectively excluded HPMRS4 in this individual and, necessarily, the digenic inheritance of PGAP2 and *PGAP3* variants. Intellectual disability syndromes are known to be heterogeneous [2] and we acknowledge that, while unlikely, variants in other genes may contribute to the specific phenotype of this individual. 

## Figures and Tables

**Table 1:** Phenotypic spectrum of patient with features of HPMRS, Mabry syndrome (MIM: 239300).

Phenotype	+/−
Hyperphosphatasia	+
Global developmental/intellectual disability	+
Delayed speech	+
Delayed age at walking/no walking	+
Hypotonia	+
Seizures	+
Brachytelephalangy	−
Tented upper lip vermillion	−
Broad nasal bridge	−
Broad nasal tip	+
Multiple congenital anomalies	−

## Data Availability

The data presented in this study are available on request from the corresponding author.
